# Editorial: What do we know about COVID-19 implications for cardiovascular disease?

**DOI:** 10.3389/fcvm.2023.1125655

**Published:** 2023-01-17

**Authors:** Zeyuan Wang, Muyun Tang, Xiaodong Luan, Hendrik Tevaearai Stahel, Masanori Aikawa, Mingxing Xie, Shuping Ge, Shuyang Zhang

**Affiliations:** ^1^Department of Cardiology, Chinese Academy of Medical Sciences and Peking Union Medical College, Beijing, China; ^2^Department of Medical Research Center, State Key Laboratory of Complex Severe and Rare Diseases, Peking Union Medical College Hospital, Chinese Academy of Medical Science and Peking Union Medical College, Beijing, China; ^3^Department of Cardiovascular Surgery, Bern University Hospital, University of Bern, Bern, Switzerland; ^4^Center for Interdisciplinary Cardiovascular Sciences, Division of Cardiovascular Medicine, Brigham and Women's Hospital, Harvard Medical School, Boston, MA, United States; ^5^Center for Excellence in Vascular Biology, Department of Medicine, Brigham and Women's Hospital, Harvard Medical School, Boston, MA, United States; ^6^Department of Ultrasound, Union Hospital, Tongji Medical College, Huazhong University of Science and Technology, Wuhan, China; ^7^Hubei Province Clinical Research Center for Medical Imaging, Wuhan, China; ^8^Hubei Province Key Laboratory of Molecular Imaging, Wuhan, China; ^9^Department of Pediatrics, St Christopher's Hospital for Children, Philadelphia, PA, United States

**Keywords:** cardiovascular disease, COVID-19, mechanism, treatment, diagnosis

Public health emergencies caused by Coronavirus-19 (COVID-19) continue to exist across the globe. Studies have shown that infection with COVID-19 is more severe in people with preexisting cardiovascular disease (1, 2). Likewise, the findings of previous clinical studies indicate that COVID-19 can lead to a wide range of cardiovascular complications (3). It has been suggested that there may be a bidirectional cause-effect relationship between COVID-19 and cardiovascular disease. Over 2 years after the initial outbreak of SARS-CoV-2, there is strong evidence that people with cardiovascular diseases are both more susceptible to severe COVID-19 and are more likely to experience post-acute sequelae (4). Given this, it's significant and urgent to the underlying mechanisms need to be clarified. The present Research Topic aims to present some of the more recent acquisitions on the integration of clinical observations and experimental findings linking COVID-19 and cardiovascular disease.

With regards to better diagnostic measures, Li et al. demonstrated the prognostic value of echocardiographic parameters in COVID-19 infections with underlying cardiovascular disease; Yu et al. found that the myoglobin level may help assess the prognosis and treatment response of COVID-19 patients.

In terms of research methodology, human induced pluripotent stem cell-derived cardiac myocytes (hiPSC-CMs) may be a practical research vehicle, Jakobi et al. evaluated the effects of five drugs used to treat COVID-19 on hiPSC-CMs, which deepened our understanding of the cardiomyocyte response to drugs. Meyer et al. then developed a systematic integrated approach to summarize the detailed mechanisms of cardiovascular complications in COVID-19. By integrating COVID-19 factors into the existing coronary heart disease (CHD) Model, and evaluating the effects of different health factors and pharmacological interventions on the severity of COVID-19, Meyer et al. explained in detail the mechanisms by which the interactions between inflammation, endothelial cell injury, hypercoagulability, and hypoxia lead to patient death, elaborating the influence of each factor on the severity of the disease, which has implications for understanding the mechanisms of this disease and guiding the clinical management. Structural biology and computer-assisted drug screening may be an emerging research approach. Al-Moubarak et al. explored the interaction between potential COVID-19 antiviral therapy and hERG potassium channel pores through computer model simulations based on the cryoelectron microscopic structure of hERG, explaining the differences in the ability of different drugs to enter the lateral binding pocket.

COVID-19 affects the cardiovascular system through various mechanisms. The SARS-CoV-2 infection can cause myocardial injury and associated with increased in-hospital mortality (5). Pathologists performed cardiac tissue autopsies on patients with COVID-19, thereby confirming the presence of myocarditis, and noted that most patients exhibited increased cardiac interstitial macrophage infiltration (6). Song et al. suggested that a high inflammatory burden might be a potential cause of myocardial injury in critically ill patients with COVID-19. In addition, COVID-19 also involves the vascular endothelium. Jud et al. suggest that it may promote COVID-19-related endothelial dysfunction and inflammatory vasculopathy by affecting arterial stiffness, capillary morphology, homocysteine metabolism, etc. Meanwhile, Jha et al. analyzed gene expression in patients' lung epithelial cells by transcriptomics and found alterations in genes related to apoptosis, coagulation, and vascular function, suggesting that these may be associated with the development of cardiovascular complications. The human receptor angiotensin-converting enzyme 2 (ACE2) dimer may be an important target for mediating viral damage to the cardiovascular system and indeed the systemic system by binding to the viral trimeric stinger protein (7). A multicenter retrospective cohort study by Huang et al. showed that angiotensin-converting enzyme inhibitors (ACEI) or angiotensin receptor blockers (ARBs) may alleviate organ damage by reducing pro-inflammatory cytokine levels, but simultaneously prolong viral shedding, so antiviral therapy should be intensified concomitantly and hemodynamic changes should be closely monitored. However, Raisi-Estabragh et al. reviewed the status of 7,099 people on UK Biobank and found that ACE/ARB use was not associated with COVID-19 status. Activation of autoantibodies and systemic inflammation in COVID-19 patients may also have cardiovascular involvement (8). Lumish et al. noted that higher levels of high-sensitivity cardiac troponin T (hs-cTNT), high-sensitivity C-reactive protein (hs-CRP), and creatinine may be associated with higher morbidity and mortality in men. Sun et al. noted that patients with severe COVID-19 have lower levels of HDL-C and apoA-1, and these may be promising predictors of severe disease and in-hospital mortality in COVID-19 patients.

COVID-19 patients with combined cardiovascular complications require particular attention in terms of treatment and prognosis. Myocardial injury with elevated plasma troponin is seen in 8–12% of COVID-19 patients (3). The studies performed by Rubattu et al. have found the potential beneficial role of angiotensin receptor II blocker - neprilysin inhibitor (ARNI) in heart failure patients with COVID-19. Petersen-Uribe et al. found that heart failure and pre-existing cardiovascular disease in COVID-19 patients are associated with serious complications such as acute respiratory distress syndrome (ARDS). Remaining in the field of cardiovascular impairment treatment during COVID-19, Wang et al. validated the key importance of anti-inflammatories to address the cardiac implications of COVID-19, especially among severe cases and critical cases. Given the large scale of the COVID-19 pandemic worldwide, cardiovascular sequelae of COVID-19 are a significant concern to all populations with worse short-term outcomes (9). Hence, physicians must be aware of these diseases and establish treatment as early as possible.

In summary, the present Research Topic indicates that advances in clinical management and mechanistic basis achieved recently with the interaction of SARS-CoV-2 infection and the cardiovascular system. Additionally, the findings discussed herein might promote awareness of these implications and stimulate further research.

## Author contributions

ZW, MT, XL, and SZ: concept and design. ZW and MT: drafting of the manuscript. HT, MA, MX, and SG: administrative and supervision. XL and SZ: critical revision of the manuscript for important intellectual content, administrative, and supervision. All authors contributed to the article and approved the submitted version.
